# Supplementary material for: Evidence that synthetic lethality underlies the mutual exclusivity of oncogenic KRAS and EGFR mutations in lung adenocarcinoma
Source: eLife. 2015 Jun 5;4:e06907. doi: 10.7554/eLife.06907 (PMC4478584; doi:10.7554/eLife.06907)
Supplement: Supplementary file 3. — Genotypes for all experimental mice used in this study (Below). DOI: http://dx.doi.org/10.7554/eLife.06907.011 [file elife06907s003.docx]

**Supplementary File 3**: Genotypes for all experimental mice used in this study

| **Mouse Number** | **CCSP-rtTA Genotype*** | **TetO-KRAS-G12D Genotype*** | **TetO-EGFR-DEL Genotype*** |
| --- | --- | --- | --- |
| 678 | + | - | + |
| 679 | + | - | + |
| 681 | + | + | + |
| 682 | + | - | - |
| 683 | ++ | + | - |
| 684 | ++ | - | - |
| 685 | + | + | + |
| 687 | ++ | + | + |
| 688 | + | + | + |
| 689 | ++ | - | - |
| 772 | + | - | - |
| 774 | + | - | + |
| 775 | ++ | + | - |
| 789 | ++ | + | - |
| 790 | ++ | + | - |
| 791 | ++ | - | + |
| 792 | ++ | - | - |
| 793 | ++ | - | - |
| 794 | + | - | + |
| 795 | ++ | + | - |
| 796 | + | - | - |
| 797 | + | + | + |
| 798 | ++ | + | + |
| 799 | ++ | + | - |
| 847 | + | + | - |
| 848 | ++ | + | + |
| 849 | ++ | - | - |
| 850 | + | - | + |
| 851 | ++ | - | + |
| 853 | + | - | - |
| 902 | ++ | - | + |
| 903 | + | + | + |
| 904 | + | + | - |
| 905 | ++ | - | - |
| 906 | ++ | + | - |
| 907 | + | - | - |
| 908 | + | - | - |
| 909 | ++ | - | + |
| 1105 | ++ | - | - |
| 1106 | + | - | - |
| 1107 | ++ | - | + |
| 1108 | + | - | + |
| 1109 | ++ | + | + |
| 1110 | + | - | + |
| 1111 | ++ | - | + |
| 1112 | + | + | - |
| 1113 | + | - | - |
| 1114 | ++ | - | - |

***** + Hemizygous, ++ Homozygous, - Negative
