# Supplementary material for: Evidence that synthetic lethality underlies the mutual exclusivity of oncogenic KRAS and EGFR mutations in lung adenocarcinoma
Source: eLife. 2015 Jun 5;4:e06907. doi: 10.7554/eLife.06907 (PMC4478584; doi:10.7554/eLife.06907)
Supplement: Supplementary file 5. — Gene sets enriched upon co-induction of mutant KRAS and EGFR. DOI: http://dx.doi.org/10.7554/eLife.06907.013 [file elife06907s005.docx]

**Supplementary File 5:** Gene sets enriched upon mutant KRAS and EGFR co-induction.

| **Gene Set Rank** | **Gene Set Name*** | **Gene Set Description** | **Gene Set Size** | **Enrichment Score** | **Normalized Enrichment Score** | **p-value** | **FDR**  **q-value** | **FWER p-value** | **Rank at Max** | **Leading Edge** |
| --- | --- | --- | --- | --- | --- | --- | --- | --- | --- | --- |
| 1 | KRAS.600.LUNG.BREAST_UP.V1_UP | Genes upregulated in epithelial lung and breast cancer cell lines overexpressing an oncogenic form of KRAS | 14 | 0.64 | 2.29 | 0 | 0 | 0 | 30 | tags=71%, list=23%, signal=83% |
| 2 | KRAS.LUNG.BREAST_UP.V1_UP | Genes upregulated in epithelial lung and breast cancer cell lines overexpressing an oncogenic KRAS gene | 11 | 0.65 | 2.16 | 0 | 0 | 0 | 45 | tags=91%, list=34%, signal=126% |
| 3 | STK33_UP | Genes upregulated in NOMO-1 and SKM-1 cells (AML) after knockdown of STK33 by RNAi | 10 | 0.63 | 2 | 0.002 | 0.005 | 0.008 | 45 | tags=90%, list=34%, signal=126% |
| 4 | STK33_NOMO_UP | Genes upregulated in NOMO-1 cells (AML) after knockdown of STK33 by RNAi | 10 | 0.58 | 1.85 | 0.002 | 0.007 | 0.017 | 50 | tags=90%, list=38%, signal=134% |

*****Additional details on gene set sources can be found at http://www.broadinstitute.org/gsea/msigdb
